# Cysteine modifiers suggest an allosteric inhibitory site on the CAL PDZ domain

**DOI:** 10.1042/BSR20180231

**Published:** 2018-07-06

**Authors:** Yu Zhao, Patrick R. Cushing, David C. Smithson, Maria Pellegrini, Alexandre A. Pletnev, Sahar Al-Ayyoubi, Andrew V. Grassetti, Scott A. Gerber, R. Kiplin Guy, Dean R. Madden

**Affiliations:** 1 Department of Biochemistry and Cell Biology, Geisel School of Medicine at Dartmouth, Hanover, NH 03755, U.S.A.; 2 Department of Chemical Biology and Therapeutics, St. Jude Children’s Research Hospital, Memphis, TN 38105, U.S.A.; 3 Department of Chemistry, Dartmouth College, Hanover, NH 03755, U.S.A.; 4 Department of Molecular and Systems Biology, Geisel School of Medicine at Dartmouth, Hanover, NH 03755, U.S.A.; 5 PRC, LakePharma, Worcester, MA, U.S.A.; 6 DCS, Genentech, South San Francisco, CA, U.S.A.; 7 SAA, Navigant, New York City, NY, U.S.A.; 8 RKG, School of Pharmacy, University of Kentucky, Lexington, KY, U.S.A

**Keywords:** covalent allostery, high-throughput screen, NMR spectroscopy, protein-protein interactions, X-ray crystallography

## Abstract

Protein–protein interactions have become attractive targets for both experimental and therapeutic interventions. The PSD-95/Dlg1/ZO-1 (PDZ) domain is found in a large family of eukaryotic scaffold proteins that plays important roles in intracellular trafficking and localization of many target proteins. Here, we seek inhibitors of the PDZ protein that facilitates post-endocytic degradation of the cystic fibrosis (CF) transmembrane conductance regulator (CFTR): the CFTR-associated ligand (CAL). We develop and validate biochemical screens and identify methyl-3,4-dephostatin (MD) and its analog ethyl-3,4-dephostatin (ED) as CAL PDZ inhibitors. Depending on conditions, MD can bind either covalently or non-covalently. Crystallographic and NMR data confirm that MD attacks a pocket at a site distinct from the canonical peptide-binding groove, and suggests an allosteric connection between target residue Cys^319^ and the conserved Leu^291^ in the GLGI motif. MD and ED thus appear to represent the first examples of small-molecule allosteric regulation of PDZ:peptide affinity. Their mechanism of action may exploit the known conformational plasticity of the PDZ domains and suggests that allosteric modulation may represent a strategy for targeting of this family of protein–protein binding modules.

## Introduction

Protein–protein interactions play crucial roles in many biological processes, both normal and pathological. Therefore, they are attractive targets for both experimental and therapeutic interventions. However, such interactions can be difficult to disrupt, due to their distributed contact surfaces and shallow pockets. In the past two decades, much effort has been devoted to understand such interactions and targeting them therapeutically. Success has been achieved by targeting protein binding sites directly. Complex chemistries such as linear peptides, macrocycles, or more complex secondary and tertiary structures can mediate the relatively extensive interactions that are needed to disrupt the binding interface [1,2].

One class of interactions, mediated by the PSD-95/Dlg1/ZO-1 (PDZ) domains first observed in the proteins post-synaptic density 95 (PSD-95), discs large 1 (Dlg1), and zonula occludens 1 (ZO1) [3,4], typically engages peptides carrying short linear motifs (SLiMs). PDZ domains have been found in more than 150 human proteins. They are involved in protein trafficking and localization, signal transduction pathways, and intercellular communications [5–7]. The canonical fold consists of two or three α-helices and five or six β-strands. Peptide ligands bind in an extended conformation in a shallow, exposed cleft formed by the α2 helix, the β2 strand, and a carboxylate-binding loop [8]. Due to the relatively conserved binding mode and their roles in mediating a wide variety of physiologically important interactions, PDZ domains have emerged as attractive targets for inhibitor development [6]. In addition to the complex chemistries, a number of small molecules have been developed to inhibit different PDZ domains, all of which target the peptide-binding cleft either competitively or through a covalent attachment [9–13].

Canonical PDZ interactions play important roles in the intracellular trafficking of cystic fibrosis (CF) transmembrane conductance regulator (CFTR). CFTR is an epithelial ion channel that is mutated in patients with CF, a lethal genetic disease that affects roughly 70000 patients worldwide [14]. The most common variant allele is c.1521_1523delCTT (*F508del-CFTR*), encoding protein p.Phe508del (F508del) in which residue Phe^508^ is deleted. This mutation impairs protein biogenesis, function, and half-life. While a number of modulator therapies are emerging [15–18], selective PDZ modulation can boost efficacy of these combinations. Specifically, we have shown that the CFTR-associated ligand (CAL), a CFTR trafficking PDZ protein, negatively regulates the apical half-life and functional activity of CFTR at the apical membrane in CF patient-derived bronchial epithelial cells expressing either *F508del-* or *WT-CFTR* (Supplementary Figure S1). As a result, CAL PDZ inhibitors can enhance rescue of apical CFTR abundance [19,20].

Previous studies inhibited the PDZ–CFTR interaction using either CAL-specific siRNA or peptide inhibitors, providing proof-of-concept of the benefits of inhibiting CAL [19,21–24]. In the present study, we described the development and initial performance of a high-throughput screening platform that identified several candidate small-molecule inhibitors of the CAL PDZ domain. We also reported the stereochemical validation of one inhibitor scaffold and its interaction with CAL, which suggested a mechanism of small-molecule inhibition not previously seen for PDZ domains.

## Materials and methods

### Cloning of expression constructs

Cerulean fluorescent protein (a gift from Dr Nancy Speck, University of Pennsylvania) was PCR amplified and subcloned into a pET16b vector that expresses CAL (UniProt accession #Q9HD26-2) PDZ domain (CALP) with an N-terminal poly-histidine tag (CALP-His_10_) [20] via NcoI and NdeI restriction enzyme sites, yielding a Cerulean-CALP fusion (Cer-CALP).

A plasmid for expression of the CALP^C319A^ site-directed mutant was generated from pET16b-CALP, which expresses CALP with a cleavable poly-histidine tag [25], using the QuikChange Lightning Site-Directed Mutagenesis Kit (Stratagene).

### Protein purification and mutagenesis

CALP and CALP^C319A^ [25] and CALP-His_10_ [20] proteins were expressed and purified as previously described. Cer-CALP vector was transformed into BL21(DE3) RIL cells and protein was expressed essentially as described for Na^+^/H^+^ Exchanger Regulatory Factor (NHERF)1 PDZ1 [21]. Briefly, cells were induced for ~16 h at 20°C and harvested by centrifugation. Following lysis by French press, the lysate was clarified by ultracentrifugation and purified using a HisTrap nickel column (GE Healthcare), and then further purified using size-exclusion chromatography (SEC) with a Superdex S75 column (GE Healthcare) equilibrated in 25 mM Tris (pH 8.5), 150 mM NaCl, 0.1 mM tris(2-carboxyethyl)phosphine (TCEP), 0.1 mM ATP, 0.02% (*w/v*) NaN_3_. The protein was dialyzed into 25 mM sodium phosphate (pH 7.4), 150 mM NaCl, 0.1 mM TCEP, and 5% (*v/v*) glycerol for cryostorage.

### Peptide synthesis

All peptides were synthesized by Tufts, Keck, or St. Jude peptide-synthesis core facilities. F*-, TMR*-, and *BT-* prefixes indicate peptides with N-terminal fluorescein or 5(6)-carboxytetramethylrhodamine coupled via an amino-hexanoic acid linker or biotin (BT) coupled directly to the N-terminus, respectively. Lowercase letters in reference to peptide sequences represent d-amino acids.

### High-throughput assay automation

All screening data were generated on a High Resolution Engineering integrated screening system using Liconic plate incubators and a Stabuli T60 robotic arm. Assay solutions were dispensed using Matrix Wellmates. Plates were centrifuged after protein/reporter solution additions using a V spin plate centrifuge. Compound transfers were performed using a 384-well pin tool equipped with 10 nl slotted hydrophobic surface-coated pins. For all assays, the positive control was the CALP peptide inhibitor iCAL23 (WrFKKANSRWPTSII), and the negative control was an equal volume of DMSO. Screening hits were identified on a plate-by-plate basis by calculating inhibitor cutoffs equal to the first quartile minus 1.5 times the interquartile range.

### Cer-CALP:TMR*-iCAL36 FRET screen

Cer-CALP and TMR*-iCAL36 (ANSRWPTSII) were incubated at equimolar concentrations of 2.15 µM in HTS buffer (25 mM sodium phosphate (pH 7.4), 150 mM NaCl, 0.1 mM TCEP, 0.5 mM Thesit, 0.1 mg/ml bovine IgG) for at least 30 min, and then 25-µl aliquots were transferred to microplates. Compounds and controls were added, microplates were centrifuged, and the solutions were allowed to equilibrate at room temperature for 1 h. The FRET ratio was determined by measuring the ratio of TMR* to Cerulean fluorescence emission at 575 nm and 475 nm, respectively, following excitation of Cerulean at 425 nm.

### Cer-CALP:*BT*-iCAL38 AlphaScreen

Cer-CALP (0.42 µM) and *BT*-iCAL38 (WrFKKfNSRWPTSII; 1.0 µM) were mixed for 30 min in AlphaScreen (AS) buffer (HTS buffer without sodium azide). Fifteen microliters of protein and peptide solution was transferred to microplates. Compounds and controls were subsequently transferred to the microplates and were allowed to incubate for 30 min followed by 10 µl nickel chelate and streptavidin AS beads, yielding a final concentration of 20 µg/ml beads with 0.25 µM Cer-CALP and 0.6 µM *BT*-iCAL38. Solutions were incubated for another 45 min prior to measurement of emission from 520–620 nm upon excitation at 680 nm in an EnVision microplate reader (Perkin Elmer).

### Fluorescence polarization

Fluorescence polarization (FP) experiments were performed as previously described [21]. For *K*_D_ measurements, 30 nM reporter ligand (F*-iCAL36) was incubated with serial dilutions of protein for 30 min at room temperature before measurement of fluorescence anisotropy data. Protein was diluted in FP buffer (25 mM sodium phosphate, 150 mM sodium chloride, 0.02% (*w/v*) sodium azide, 0.1 mM TCEP containing 0.1 mg/ml IgG and 0.5 mM Thesit). For *K*_I_ measurements, 30 nM of the same reporter ligand was incubated with protein at a concentration of 1.8*K_D_ for 30 min at room temperature. An aliquot of 38 µl of this protein:reporter mixture was then incubated with 2 µl of serial dilution of the inhibitor for 30 min at room temperature before measurement. Titrations were fit as previously described [21].

### Apparent *K*_D_ measurements for modified proteins

Aliquots of 50 µM CALP or CALP^C319A^ were incubated separately with one of three small molecules at a concentration of 500 µM for ~4 h at RT: methyl-3,4-dephostatin (MD), ethyl-3,4-dephostatin (ED), or *N*-ethyl-maleimide (EM). Excess small-molecule label was removed through dialysis (two 2-h buffer exchanges). The dialyzed proteins were immediately used in a FP binding assay, and the *K*_D_ was determined, as previously described [21]. The buffer used for incubation, dialysis, and FP measurements contained 25 mM sodium phosphate, 150 mM sodium chloride, and 0.02% (*w/v*) sodium azide (labeling buffer) at pH 6.8. The extent of small-molecule adduct formation was quantitated by integrating the peaks observed by MALDI-TOF MS at the corresponding unlabeled and labeled molecular weights. Covalent protein dimerization was assessed by non-reducing SDS/PAGE. A preliminary set of labeling experiments was performed for ~48 h at 4°C, using 243 µM protein and 270 µM compound, and 25 mM Tris(hydroxymethyl)aminomethane (pH 9) instead of sodium phosphate; dialysis was performed overnight in labeling buffer at pH 7.4.

### CALP isotopic labeling

CALP was isotopically labeled either with ^15^N or with ^15^N and ^13^C as follows. BL21(DE3) RIL cells were grown overnight at 37°C in 2×YT. To initiate labeling, cells were washed twice by centrifugation, decantation, and resuspension in minimal medium. Cells were then diluted 100:1 in minimal medium and grown at 37°C. Lysis and purification were performed as described for unlabeled CALP [20,25]. Minimal medium contained, in addition to 0.1 mg/l ampicillin, 0.037 mg/l chloramphenicol, 1× BME vitamins (Sigma), and 5 mg/l thiamine (in mM): 50 KH_2_PO_4_, 30 Na_3_C_6_H_5_O_7_*2H_2_O, 0.7 CaCl_2_*2H_2_O, 10 MgSO_4_*7H_2_O, 1 FeCl_3_*6H_2_O, 0.09 ZnCl_2_, 0.12 CoCl_2_*6H_2_O, 0.075 CuCl_2_*2H_2_O, 0.083 Na_2_MoO_4_*2H_2_O, 0.068 CaCl_2_*2H_2_O, 0.0745 H_3_BO_3_, 0.12 HCl. Minimal medium also contained 3 g/l ^15^N-NH_4_Cl and 10 g/l glucose for expression of ^15^N-CALP or 3 g/l ^15^N-NH_4_Cl and 4 g/l ^13^C-glucose (Cambridge Isotope Laboratories) for expression of ^13^C/^15^N-CALP. ^15^N-CALP-His_10_ was expressed and purified as previously described [20,21].

### NMR spectroscopy

NMR experiments were conducted at 25°C on a Bruker 600 MHz spectrometer, equipped with a TCI cryogenic probe. Two CAL PDZ constructs were used for NMR: CALP-His_10_ [21] and tag-free CALP [25]. ^15^N-CALP-His_10_ was used only for the initial validation of the 12 FRET^+^/AS^+^ candidates (Supplementary Figure S2). Backbone assignments for tag-free CALP were achieved using 755 µM protein through 2D ^1^H-^15^N heteronuclear single quantum coherence (HSQC) and 3D HNCA, HN(CO)CA, HNCACB, and CBCA(CO)NH experiments following dialysis into 25 mM sodium phosphate pH 6.8, 50 mM NaCl, 0.1 mM TCEP, 0.02% NaN_3_ (NMR buffer). Eighty-one of eighty-two non-proline residues were assigned. All NMR HSQC binding spectra with CALP were collected with 50 µM protein. iCAL1155, MD, and iCAL1113 dose–response measurements were conducted with 5% (*v/v*) D_6_-DMSO throughout. Due to slow exchange, fractional occupancy was determined by integrating cross-peaks for a given residue at both the first bound and unbound positions as a function of ligand concentration using Sparky, and then dividing the bound value by the sum. Fractional occupancy values at each concentration were averaged for at least six residues, and the resulting titration data were fit by a four-parameter logistic curve to determine EC_50_. For small-molecule footprinting, chemical-shift perturbations (Δ) were normalized using Δ = √((Δ^1^H)^2^ + (Δ^15^N/6)^2^); cross-peaks that exhibited perturbations 3*S.D.> (Δ) ≥2*S.D. were labeled orange, and (Δ) ≥3*S.D. were labeled red in (Figure 2B,E,H). Compound footprints were visualized using a model of the structure of the CAL PDZ domain (PDB ID 2LOB) using PyMOL [26].

### Crystallization, data collection, and processing

Crystals were obtained and diffraction data were collected and processed as previously described [25,27]. For co-crystallization, 5 mg/ml CALP was supplemented with 1 mM peptide HPV18E6 (RLQRRRETQV) and 1 mM MD at 2% (*v/v*) DMSO final concentration. For mutant co-crystallization 5 mg/ml CALP^C319A^ was supplemented with 1 mM peptide HPV18E6, and after crystals had formed, MD was added to the drop at ~10 mM with 10% (*v/v*) DMSO final concentration. For the CALP co-crystal, 0.3° oscillation images were collected over 360° at 100 K at the X6A beamline of the National Synchrotron Light Source at Brookhaven National Laboratory. For the CALP^C319A^ co-crystal, 0.3° oscillation images were collected over 210° at 100 K at the beamline 14-1 of the Stanford Synchrotron Radiation Lightsource (please refer to Table 1 for more information). XDS [28] was used to process the diffraction images. The crystal structure of the CALP:iCAL36 complex (PDB ID: 4E34) was used as a molecular replacement search model. Phenix [29,30] and Wincoot [31] were used for structure refinement and PyMOL [26] was used to generate the images of the model. For the wild-type CALP structure, density for MD was seen after the second round of refinement, and MD was added to the model in the third round of refinement. As expected, no additional density was seen in the vicinity of Ala^319^ for the CALP^C319A^ crystals; hence MD is not present in the final model of the mutant.

**Table 1:** Data collection and refinement statistics

	CALP + MD + HPV18E6	CALP^C319A^ + HPV18E6
**Data collection**		
Wavelength (Å)	0.9795	0.9795
Space group (number)	*P* 2_1_ (4)	*P* 2_1_ (4)
Unit cell dimensions:		
*a, b, c* (Å)	32.8, 48.3, 51.6	32.7, 48.3, 51.4
α, β, γ (°)	90.0, 101.5, 90.0	90.0, 101.8, 90.0
Resolution (Å)	19.29–1.70 (1.81–1.70)*	18.79–1.36 (1.46–1.36)*
R_sym_^†^ (%)	6.2 (68.6)*	5.3 (61.8)*
I/σ_I_	23.78 (3.34)*	17.23 (3.09)*
Completeness (%)	99.7 (99.5)*	99.1 (99.1)*
Redundancy	7.51 (7.51)*	4.26 (4.26)*
**Refinement**		
Total reflections	17472	33527
Test-set reflections	858	1700
*R*_work_^‡^/*R*_free[Table-fn T1TFN3]_^^§^^ (%)	17.4/22.6	15.4/18.3
Number of atoms:		
Protein/peptide	1457	1607
Solvent	144	143
Ligand	12	0
Ramachandran plot[Table-fn T1TFN5]^║^ (%)	99.46/0.54/0.00	99.50/0.50/0.00
*B*_av_ (Å^2^)		
Protein	23.68	22.04
Solvent	33.72	37.03
Ligand	69.63	0.00
Bond length RMSD (Å)	0.007	0.006
Bond angle RMSD (°)	1.059	0.879
PDB ID	5IC3	5K4F

* Values in parentheses are for data in the highest resolution shell.

† *R*_sym_ = Σ*_h_*Σ_i_|*I*(*h*) – I*_i_*(*h*)|/Σ*_h_*Σ*_i_ I_i_*(*h*), where *I_i_*(*h*) and *I*(*h*) values are the *i*-th and mean measurements of the intensity of reflection *h*.

‡ *R*_work_ = Σ*_h_*|*F*_obs_(*h*) – *F*_calc_(*h*)|/Σ*_h_ F*_obs_(*h*), *h* ∈ {working set}.

§ *R*_free_ = Σ*_h_*|*F*_obs_(*h*) – *F*_calc_(*h*)|/Σ*_h_ F*_obs_(*h*), *h* ∈ {test set}.

║ Favored/allowed/outliers.

### CAL knockdown and CFTR surface biotinylation

CFBE 41o- CF bronchial epithelial cells expressing WT CFTR (CFBE-WT cells) or F508del (CFBE-ΔF cells) [32] were a generous gift from Dr J.P. Clancy (University of Alabama, Birmingham). CFBE-WT cells were treated with CAL-specific (siCAL, 200 nM) or control (siNEG, 200 nM) RNAi mixed with HiPerFect (Qiagen) transfection agent and Opti-MEM (Gibco) and seeded on plastic dishes for 24 h. The next day, cells were harvested and reseeded on 4.67 cm^2^ surface area and 0.4 μm pore size Transwell permeable supports (Corning) coated with collagen. Apical medium was removed 24 h post seeding, and cells were grown at air–liquid interface for 3 days prior to surface biotinylation.

Transwell plates were kept on ice throughout the surface-biotinylation procedure. Filters were washed with cold phosphate buffer saline (Gibco), PBS ++, pH 7.4 (PBS supplemented with 1 mM MgCl_2_ and 0.1 mM CaCl_2_). PBS++ was added to the basolateral side of the filter while 0.5 ml NHC-LC-BT dissolved in PBS++, pH 7.4 (1 mg/ml; Thermo Scientific) was added to the apical surface. Plates were incubated in the dark at 4°C for 1 h. Filters were then washed on both sides with PBS++, pH 7.4 and incubated with 500 µl BLB/1XC (25 mM HEPES, pH 8.0, 1% (*v/v*) Triton X-100, 10% (*v/v*) glycerol, and Complete protease inhibitor (Roche) for 15 min on a shaker at 4°C. Cells were scraped and lysates passed through a 27-gauge half-inch needle before spinning in a Biofuge (Heraeus) at 13000 rpm at 4°C for 15 min. For surface-biotinylated proteins, the lysate was incubated with washed streptavidin beads and incubated at 4°C for 2 h. Samples were washed with BLB/1XC (25 mM HEPES, pH 8.0, 1% (*v/v*) Triton X-100, 10% (*v/v*) glycerol, and Complete protease inhibitor (Roche)) for 15 min on a shaker at 4°C. Bound protein was eluted with Laemmli sample buffer at 85°C for 3 min. Samples were loaded on SDS/PAGE (7.5% gels) and blotted on to PVDF membranes; the blots were probed with the CFTR-specific antibody 596 (provided through the CF Foundation).

### Biocompatibility assays

CFBE-ΔF cells were used for both biocompatibility assays. Cell proliferation was determined by 3-(4,5-dimethylthiazol-2-yl)-5-(3-carboxymethoxyphenyl)-2-(4-sulfophenyl)-2H-tetrazolium, inner salt (MTS) assay (CellTiter 96® AQ_ueous_ One Solution Cell Proliferation Assay [MTS], Promega). The MTS formazan product generated in each condition was quantitated after 24 h of treatment with each compound of interest. The values were normalized to the response of a control exposed to medium only.

Cell permeability was determined by lactate dehydrogenase (LDH) assay to assess cytotoxicity (CytoTox 96™ Non-Radioactive Cytotoxicity Assay, Promega). The LDH released in each condition was quantitated after 24 h of treatment with each compound of interest. The values were normalized to the control response to 0.1% Triton X-100.

In both the MTS and LDH assays, MD and iCAL1155 introduced 0.2% (*v/v*) DMSO into the system; iCAL1113 introduced 0.5% (*v/v*) DMSO into the system.

### Data availability

Co-ordinates and structure factors have been deposited in the Protein Data Bank with accession numbers **5IC3** (CALP with HPV18E6 peptide and MD) and **5K4F** (CALP^C319A^ with HPV18E6 peptide). The NMR assignment for CALP has been deposited in the Biological Magnetic Resonance Bank (BMRB) with accession number **27338**.

## Results and discussion

### A screen for small-molecule inhibitors of CAL PDZ domain

**Figure 1:**
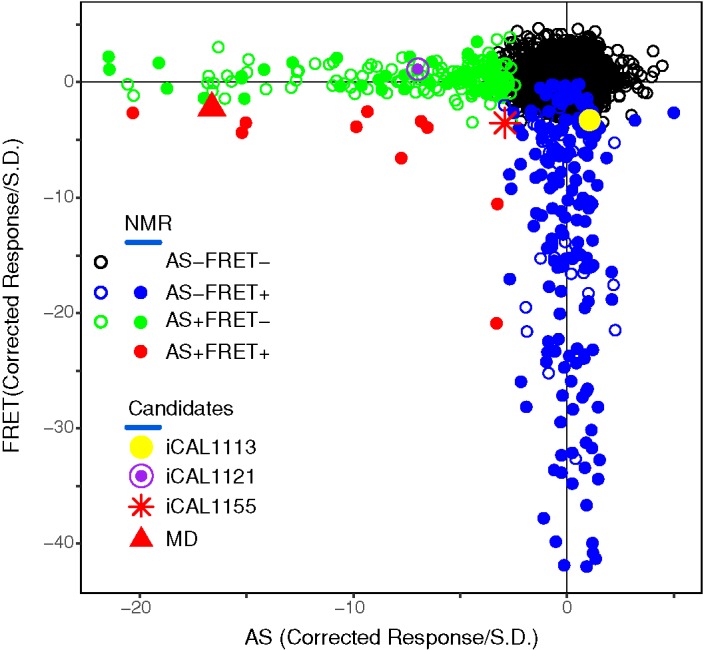
CAL inhibitor screen A scatter plot shows the AS (x-axis) and FRET (y-axis) corrected responses for each compound, excluding responses > +5σ. Plate-based metrics identified AS only (green), FRET only (blue), and AS/FRET (red) responders. HSQC spectra were determined for all double-positive and selected single-positive compounds (solid circles), and identified four candidate inhibitors (expanded icons).

To identify small molecules that could disrupt the interaction between PDZ-binding motifs and CAL, we designed high-throughput compatible assays using our peptide inhibitor iCAL36 (ANSRWPTSII) as a reporter for CALP binding [19,33]. To validate our approach, we screened a library of 5600 bioactive compounds (Figure 1), representing 3161 distinct chemistries. In preliminary experiments with carboxy-X-rhodamine- or fluorescein-labeled reporter peptides, FP competition assays exhibited relatively poor Z′ and Z values (≤0.53 and ≤0.39, respectively), and a relatively large number of the putative candidates showed significant fluorescence intensity shifts (data not shown). As a result, parallel FRET and AS proximity assays were tested as alternative primary screens.

For the FRET assay, we developed overexpression and purification protocols for a Cer-CALP fusion constructed to serve as a donor to a TMR*-labeled reporter peptide. FRET was determined by the ratio of TMR* to Cerulean fluorescence at 575 and 475 nm, respectively, following excitation of Cerulean at 425 nm. Equimolar titration of Cer-CALP and TMR*-iCAL36 revealed an EC_50_ value of 4.9 µM, consistent with the expected high-affinity protein–reporter interaction. When tested in our pilot bioactive screen in 384-well format, the Z′ and Z factors were 0.71 and 0.70, respectively. We selected candidates with FRET signals that were simultaneously outliers from the negative-control and variable-compound populations for statistical robustness [34]. The hit rate was 4.1% of wells (231/5600).

We also developed an AS assay using the Cer-CALP fusion protein and an N-terminally biotinylated reporter peptide. Titration of *BT*-iCAL38 in the presence of 0.25 µM Cer-CALP confirmed a high-affinity interaction (EC_50_ = 0.38 µM). In high-throughput screening (HTS) format with our bioactive screen, the AS assay yielded Z′ and Z factors of 0.73 and 0.75, respectively. The hit rate was 3.7% of wells (209/5600).

Both the FRET and AS assays demonstrated significantly improved Z′ and Z factors relative to our initial FP protocols. However, they both also showed relatively high hit rates. Of the 3161 unique compounds, the FRET assay identified 166 distinct compounds (5.3%; red and blue circles in Figure 1), whereas 161 were identified by AS (5.1%; red and green circles in Figure 1). Most of the hits were clustered along the respective thresholds. Nevertheless, by examining compound redundancy, we confirmed the reproducibility of the results within each assay. For example, there are 547 compounds presented twice in the library. Of the 547 duplicated compounds, 530 gave the same results in FRET assay; 539 gave the same results in AS assay. Across all replicated compounds, >95% of the decisions were ‘unanimous.’

### Validation and analysis

**Figure 2:**
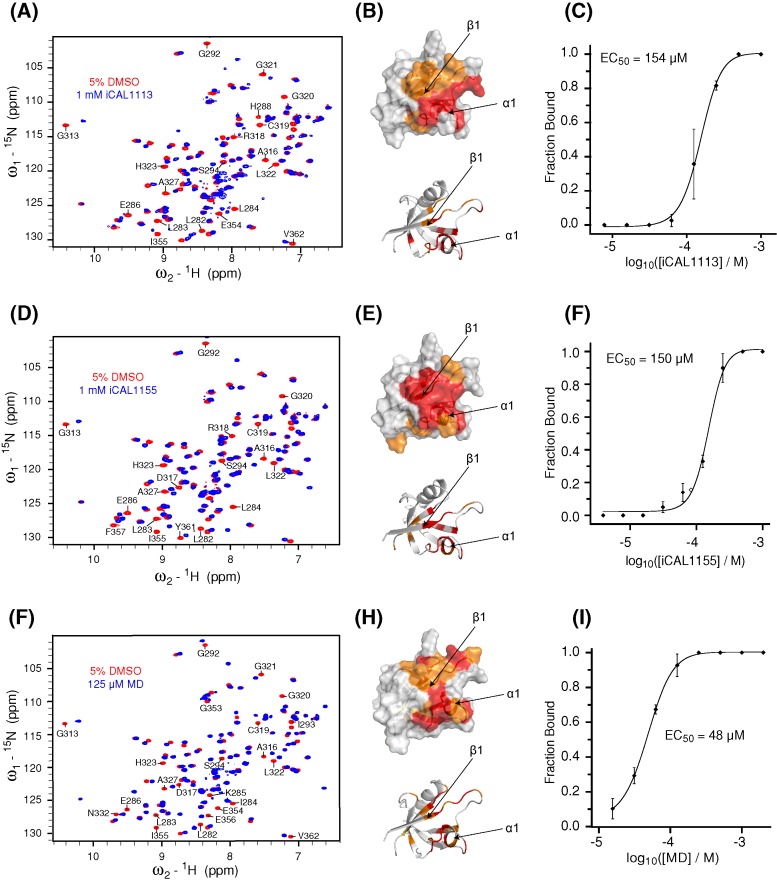
NMR HSQC footprint of candidate compounds HSQC spectra are shown for 50 μM ^15^N-CALP with either 1 mM iCAL1113 (**A**), 1 mM iCAL1155 (**D**), or 125 μM MD (**G**) in blue overlaid with control spectra collected in the presence of vehicle only (5% DMSO, red). Selected chemical-shift perturbations are identified. Significant cross-peak perturbations for iCAL1113 (**B**), iCAL1155, (**E**), and MD (**H**) after compound addition are highlighted by coloring the affected residues on the van der Waals surface (top (B,E,H) panels) of the CAL PDZ domain (PDB ID: 2LOB; orange: ≥2σ; red: ≥3σ). Arrows indicate the location of the β1 strand and α1 helix. All compounds bind in a pocket between β1 strand and α1 helix, as indicated in the cartoon model ((B,E,H) bottom panels). HSQC titrations were performed with each compound and fractional occupancy was calculated at each concentration by integrating slowly exchanging cross-peak volumes at both bound and unbound positions. Results were averaged for six cross-peaks. Averages (±S.D.) are plotted as a function of compound concentration (**C**,**F**,**I**). The EC_50_ was calculated by least-squares fitting to a logistic curve (solid line), as described in the ‘Materials and methods’ section.

Given the differences between the two assays, we initially focussed on the relatively small number of compounds (12 hits, 0.4%) that were identified in both (Figure 1, red symbols and Supplementary Figure S2). One compound fell beyond the range of the positive control and was not pursued further. Amongst the remaining 11 candidates, HSQC footprints of the CAL PDZ domain identified seven direct interactors (58% true positives). Five of these cause global changes consistent with destabilization of the protein fold. The remaining two compounds, iCAL1155 and MD, caused residue-specific chemical-shift perturbations (Figure 2D,G) and were selected for further evaluation. To assess the false-negative implications of selecting only FRET^+^/AS^+^ hits, we also tested 119 FRET^+^/AS^–^ and 30 FRET^–^/AS^+^ hits by NMR. From this much larger pool, only five compounds were validated by NMR footprinting, and only one compound from each set exhibited a site-specific footprint, underscoring the predictive value of combining methodologically distinct primary assays.

We then titrated the progression of chemical-shift perturbations in HSQC spectra to estimate an EC_50_ value for each of the confirmed inhibitors. The AS-positive compound iCAL1121 did not achieve saturation at 2 mM and was dropped from further study. The FRET-positive compound iCAL1113 and the double-positive compounds iCAL1155 and MD exhibited EC_50_ values ranging from 48 to 154 µM (Figure 2C,F,I).

**Figure 3:**
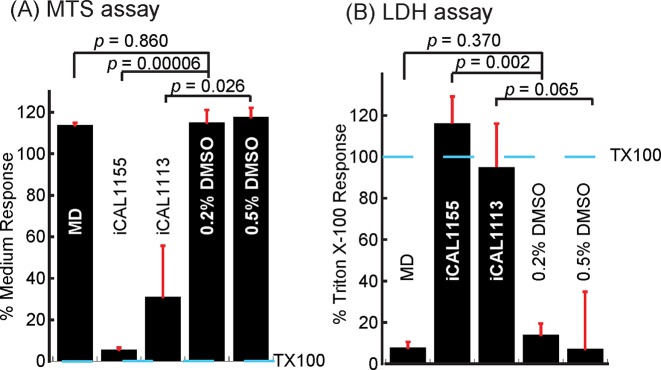
Inhibitor biocompatibility The biocompatibility of the three candidate inhibitors iCAL1113, iCAL1155, and MD was assessed using CFBE-ΔF cells. Compound concentration was set at 50 μM. (**A**) Cell proliferation assay. The values were normalized to the response of a control exposed to medium only. MD-treated cells proliferate as well as DMSO controls; iCAL1155- and iCAL1113-treated cells showed much less proliferation. (**B**) Cytotoxicity assay. The values were normalized to the control response to 0.1% Triton X-100. The toxicity of MD is comparable with DMSO control. iCAL1155- and iCAL1113-treated cells released significantly more LDH, indicating that iCAL1155 and iCAL1113 are more toxic at the same concentrations. In both assays, data represented mean ± S.E.M. Significance was tested by a two-tailed, unpaired Student’s *t* test compared with control, as indicated. TX100 marks the response to 0.1% Triton X-100.

Furthermore, while iCAL1113 and iCAL1155 cause cytotoxic and cytostatic effects when applied to CFBE-ΔF monolayers, MD exhibits relatively good cellular biocompatibility in both assays (Figure 3). We thus performed an Ussing-chamber assay to test whether MD could rescue functional F508del-CFTR. However, MD fails to stimulate CFTR chloride currents when applied to CFBE-ΔF cells (Supplementary Figure S3), which might be due to limited potency, lack of specificity, or inability to access the endocytic pathway.

Indeed, MD contains a catechol moiety and is thus probably one of the pan–assay interference compounds (PAINS) [35]. MD inhibits multiple targets, including several protein tyrosine phosphatases, such as CD45, PTP1B, and SHPTP1, as well as APOBEC3G, a cytosine deaminase with a role in the innate immune response. It also covalently modifies proteins [36–40]. Therefore, from a therapeutic perspective, MD is unlikely to represent a CF drug scaffold.

### Identifying the binding pocket of MD

**Figure 4:**
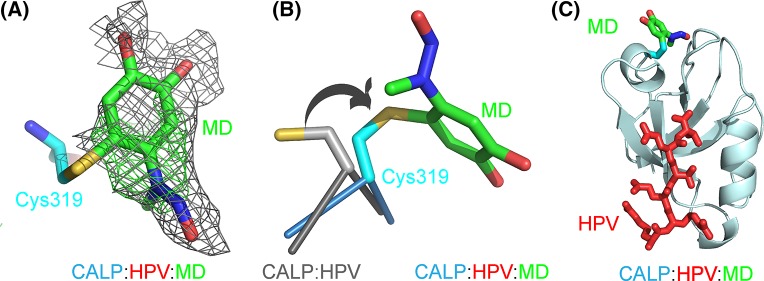
Covalent modification of CALP with MD (**A**) Positive *F*_O_ – *F*_C_ difference omit (green mesh, 2.5σ) and refined 2*F*_O_ – *F*_C_ electron density (gray mesh, 0.5σ) is observed adjacent to the Cys^319^ side chain (cyan carbons) for covalently attached MD (green carbons). (**B**) Alignment of the covalent adduct (cyan carbons) and the MD-free peptide complex (PDB ID: 4JOR; gray carbons) shows the rotation (arrow) of the Cys^319^ side chain required to accommodate the attachment of MD. (**C**) Heterotrimer structure (PDB ID: 5IC3) of CAL PDZ with a peptide (HPV18E6) and an inhibitor (MD) bound shows the spatial relationship between CALP (pale cyan), peptide HPV18E6 (red), cysteine side chain (cyan) and MD (green carbons). The non-covalent binding site of MD is in the general region of the covalent attachment site.

Nevertheless, as shown in Figure 2H, the HSQC footprint of MD revealed a binding pocket on CAL PDZ domain that is distinct from the canonical peptide-binding pocket of the protein. We therefore suspected that our screen might have identified allosteric inhibitors and wished to visualize the effect of MD binding on CALP structure and peptide-binding affinity. Unfortunately, crystallization trials with CALP and MD generated thin needle-shaped crystals that did not yield structural information. However, we were able to obtain crystals and high-resolution diffraction data for CALP in the presence of high concentrations of both MD and a low-affinity peptide partner HPV18E6 (RLQRRRETQV) [41]. Following structure determination and refinement (Table 1), we could clearly identify the PDZ domain and the bound peptide. In addition, we observed a positive peak in the difference in electron density map (Figure 4) located not in the CALP peptide-binding cleft, but rather in the vicinity of the footprint identified by NMR, consistent with an allosteric model (Supplementary Figure S4). This electron density is located on the other side of the carboxylate-binding loop that forms one end of the peptide-binding cleft and interacts with the main-chain carboxylate of the peptide. The crystallographic structure locates the MD binding site in the vicinity of Cys^319^, the only cysteine side chain in CALP. Compared with the previous MD-free structures, the cysteine side chain has rotated around its χ_1_ angle, reorienting the thiol toward the new density. In concert, the GLGI loop moves away from the peptide binding cleft by 1 Å (Supplementary Figure S5A). Otherwise, the WT and modified structures are very similar (RMSD = 0.213 Å).

### Modeling the structure of the MD–CALP complex

As mentioned above, MD has previously been shown to interact covalently with sulphydryl groups [36]. Using direct infusion ESI-Orbitrap MS, we confirmed that under crystallization conditions, a single molecule of MD covalently modifies CALP (Supplementary Figure S6A,B). We localized this modification to Cys^319^ via manual annotation of product–ion spectra.

In the MD analog ED, the –NO group of the *N*-methyl-*N*-nitroso moiety is considered a leaving group, and some studies have used ED as a nitric oxide donor to protect neurones from apoptosis [42,43]. However, loss of the nitroso moiety would result in a cysteine adduct of MD that fits poorly within the observed electron density. Furthermore, MS of both intact CALP and peptide digests following covalent modification (Supplementary Figure S6A,B) indicated that MD loses only a single proton after forming an adduct with CALP. The crystallographic structure of an APOBEC3G-MD adduct showed a covalent bond formed between the cysteine thiol and the 6 (*ortho*) position on the benzene ring of MD [36], a modification consistent with our MS data for CALP-MD. In our CALP-MD:peptide crystal structure (Figure 4), the same chemistry of attachment also yielded the best fit to the experimental electron density. The corresponding MD moiety is thus included in the refined structure.

### Covalent and non-covalent interactions with CALP

CALP–MD adduct formation is highly sensitive to pH, temperature, time, and concentration. According to MALDI-TOF results, Cys^319^ was extensively modified (>85%, *n*=3) under crystallization conditions, but much less strongly modified (≤13%, *n*=3) under NMR conditions at MD concentrations ≤125 µM or FRET screening conditions (Supplementary Figure S6C–E).

**Figure 5:**
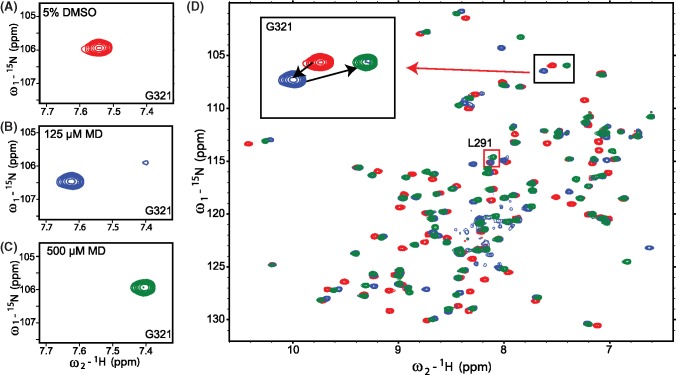
NMR HSQC titration of MD HSQC spectra are shown for 50 μM ^15^N-CALP with either 125 μM (blue) or 500 μM MD (green) overlaid with control spectra collected in the presence of vehicle only (5% DMSO, red). Two-step movement was observed for several residues across the titration from 15 to 500 μM. For example, the G321 peak (**A**) shifted upfield at 125 μM (**B**), and then downfield at 500 μM (**C**). The overlaid full spectra are shown in (**D**), with G321(black box) and L291 (red box) highlighted.

NMR titration data support the hypothesis that MD can interact with CALP both covalently and non-covalently, acting in both cases at a site near Cys^319^. The progressive disappearance of cross-peaks corresponding to the unbound state of CAL PDZ and the appearance of new cross-peaks for a subset of residues indicates that the binding is in slow exchange in the NMR timescale, consistent with an affinity in the low-to-mid micromolar range [44]. This effect appears at 15 µM MD and reaches saturation at 125 µM, where the signals from the unbound state are almost absent. Interestingly, at still higher concentrations, a new set of cross-peaks appear for some of the initially perturbed residues, corresponding to a second bound state (Figure 5). In parallel, cross-peaks corresponding to the first bound state start to decrease in intensity. The second bound state appears at 250 µM and saturates at 1 mM. From 250 µM on, only the two perturbed states are observed, with the second progressively replacing the first. Consistent with the dose–response of this second transition, when we track the modification status of CALP under NMR conditions by MALDI-TOF, we observe significant formation of CALP-MD adducts starting at 250 µM and increasing in a dose-dependent fashion (Supplementary Figure S7).

It is worth noting that most of the cross-peaks involved in the first bound state subsequently transition as a group to the second bound state, presumably reflecting binding to a similar site on CAL PDZ but via a different binding modality. Taken together, these observations are consistent with a model in which MD initially non-covalently docks to the CAL PDZ domain and subsequently forms a covalent bond. Interestingly, the chemical shifts associated with Arg^318^ and Leu^291^ exhibit much larger perturbations (>2σ) in the second bound state than in the first. For Arg^318^, this likely reflects the fact that it is adjacent to Cys^319^. However, the change at Leu^291^ cannot be accounted for by a local perturbation: it is located 10.5 Å away at the other side of the protein.

### Role of Cys^319^ in CALP inhibition

To assess the relative importance of the covalent and non-covalent interactions on MD’s ability to inhibit peptide binding, we sought to eliminate the target thiol moiety and thus prevent the covalent interaction with MD, while preserving the non-covalent affinity. We mutated Cys^319^ to alanine (CALP^C319A^) and performed an HSQC titration with ^15^N-CALP^C319A^. However, no chemical shift perturbations were detected up to an MD concentration of 500 µM (Supplementary Figure S8). At higher concentrations, precipitation was observed. Since chemical-shift perturbations of WT CALP were seen at concentrations as low as 15 µM MD, it is likely that both the covalent and non-covalent interactions between MD and CAL PDZ are strongly perturbed by the C319A mutation.

We also co-crystallized CALP^C319A^ and peptide HPV18E6, with ~10 mM MD added to the drop after crystallization, and determined the structure of the resulting complex (Table 1; PDB ID: 5K4F). Electron density is observed only for the PDZ domain and the peptide. We did not observe any electron density that might correspond to MD in the CALP^C319A^:MD:peptide structure, consistent with the loss of both non-covalent and covalent interactions seen by NMR. The C319A mutation does not affect the overall structure of the PDZ domain (Supplementary Figure S5B, RMSD = 0.213 Å). Curiously, the GLGI loop showed the same movement that was observed in the CALP:MD:peptide crystal structure (Supplementary Figure S5B,C). Therefore, the physical displacement is unlikely to represent the mechanism for allosteric cross-talk between the inhibitor and peptide-binding sites, associated with the covalent modification of MD.

### The chemistry of MD modification

**Figure 6:**
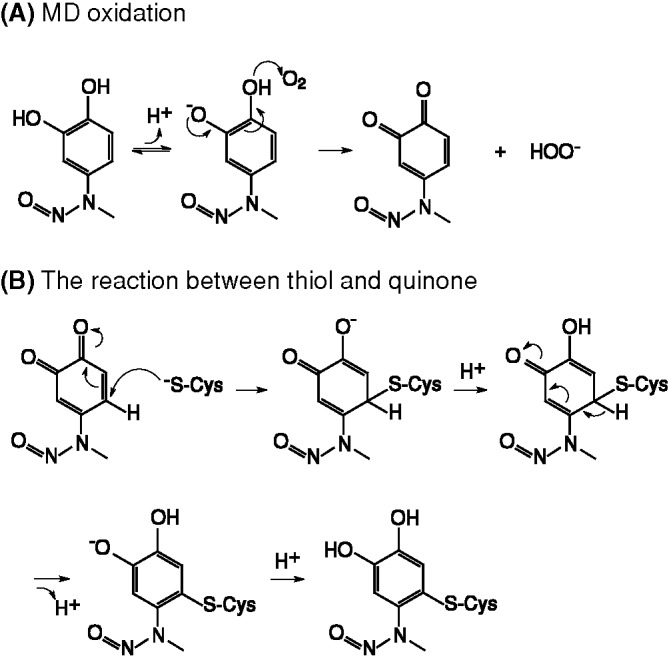
The schematics of MD oxidation (**A**) MD can be oxidized from the catechol form to the quinone form; (**B**) the quinone form of MD can react with cysteine thiol and return to the more stable catechol form after the nucleophilic attack is complete.

**Figure 7:**
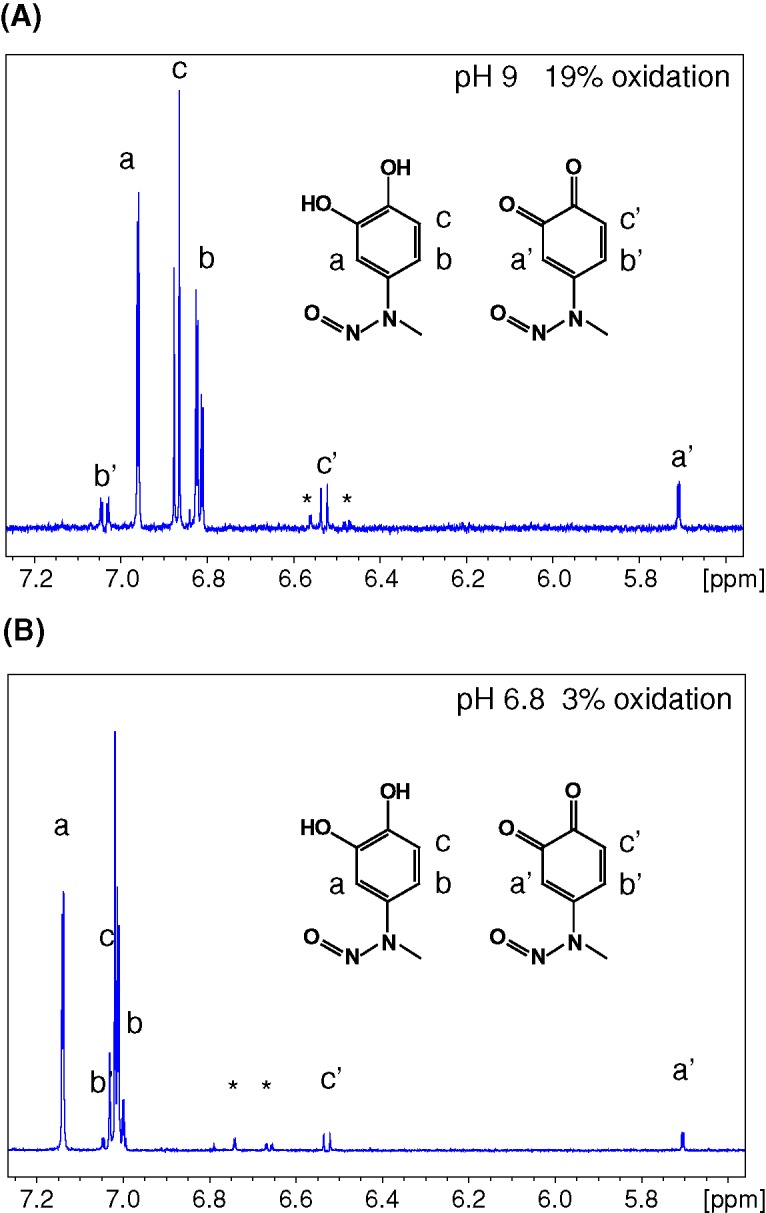
More MD oxidation was observed at higher pH in proton NMR spectra The *ortho* and *meta* protons of the benzene ring of MD have different chemical shifts between the catechol (a, b, c) and the quinone (a’, b’, c’) forms. MD (1 mM) was dissolved in buffer containing either 10 mM sodium phosphate pH 6.8 (**A**) or Tris pH 9 (**B**), 150 mM NaCl, 1% DMSO-D6 and 1% D_2_O. 1D-proton spectra of MD in different buffers were collected at 298 K. At pH 9, 19% of the total MD was observed in the oxidized quinone form according to the integrated proton peak area. However, only 3% of the total MD was oxidized to the quinone form at pH 6.8.

The mutagenesis strategy did not allow us to isolate the non-covalent mode of interaction of MD with the CAL PDZ domain. As an alternative, we sought to understand and exploit the chemistry by which MD can covalently modify cysteine thiol groups, which has not been previously described in detail. As described above, based on our MS and crystallographic data, the –NO leaving group is not involved, so the mechanism presumably involves an attack on the 6-carbon of the ring, which is unlikely in the catechol form. However, MD can undergo a redox reaction that allows the moiety to shift between catechol, semiquinone radical, and *ortho*-benzoquinone states [45]. Since MD has a relatively electron-rich arene, we hypothesized that MD may be oxidized to its quinone form (Figure 6A) before undergoing a nucleophilic attack by the cysteine thiol (Figure 6B). Such redox interconversions are also hallmarks of certain PAINS compounds [35]. We hypothesized that higher pH would promote the loss of a proton on one of the hydroxyl groups, favoring oxidation of the catechol by molecular oxygen to yield the quinone form (Figure 6A). In ^1^H NMR spectra of MD in aqueous solution (Figure 7), we observed a primary set of resonances associated with the catechol form and a secondary set of resonances consistent with the quinone form [46]. At pH 6.8, we observed only 3% of MD in the quinone form, compared with 19% in the quinone form at pH 9. Based on this mechanism, reducing agents should also be able to prevent MD from covalently modifying cysteines, by inhibiting oxidation of the MD catechol to a quinone [47].

### Inhibitory effect of covalent compared with non-covalent modifications

As a result, we can leverage the chemistry of the FP binding assay, which is compatible with 100 µM TCEP in the buffer, to suppress the covalent modification of CALP (Supplementary Figure S6D), and thus explore the impact of the non-covalent interaction. Under these conditions, FP displacement titrations showed no inhibitory effect of MD (Supplementary Figure S9A). They also showed no displacement by the close chemical analog ED nor by the common, unrelated cysteine modifier EM (Supplementary Figure S9A), included as a control for the stereochemical specificity of the modification required at Cys^319^.

As a next step, to investigate the impact of the covalent modification, we labeled CALP with either MD, ED, or EM, and then used dialysis to remove excess compound. We then performed MALDI-TOF, to confirm the extent of modification, and FP titrations, to determine apparent *K*_D_ values. Initial experiments under stringent labeling and dialysis conditions strongly favored adduction, but also led to the formation of reversible covalent PDZ dimers in the presence of the dephostatin compounds, confounding analysis of the FP data. Using less aggressive conditions and relatively rapid dialysis, we were able to obtain substantial covalent modification (>75%; Supplementary Figure S9D–F) with much lower levels of PDZ dimer formation in the presence of MD or ED (<16%). This approach yielded reproducible FP titration curves. The resulting apparent equilibrium dissociation constants reflect the impact of covalent small-molecule modification on peptide affinity.

The MD-modified domain (CALP–MD) showed a 1.9-fold loss of apparent affinity compared with the DMSO-treated control protein. The ED-modified domain (CALP–ED) showed a similar 1.9-fold loss of apparent affinity. Both of these offsets were statistically significant. In comparison, EM-modified CALP (CALP–EM) showed no significant affinity change compared with the DMSO-treated protein, confirming the importance of the dephostatin scaffold in the observed inhibition (Supplementary Figure S9B and Table 2).

**Table 2:** *K_D_* calculations for native and modified CAL PDZ

	DMSO	MD	ED	EM
**CALP**				
* K*_D_ (μM)	1.29 ± 0.16	2.43 ± 0.36	2.45 ± 0.42	1.22 ± 0.17
*P*-value compared with CALP (DMSO)		0.0001	0.0001	0.60
% covalent modification		79–81%	76–81%	90–92%
**CALP^C319A^**				
* K*_D_ (μM)	1.56 ± 0.30	1.51 ± 0.22	1.46 ± 0.21	1.51 ± 0.15
* P*-value compared with CALP^C319A^ (DMSO)		0.48	0.18	0.48
% covalent modification		0%	0%	7–9%
**Chemical structures**		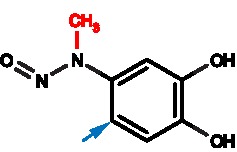	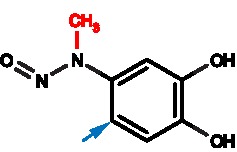	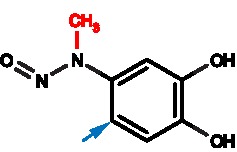

*K*_D_ values are mean ± S.D.

Schematics show the chemical structures of MD, ED, and EM. The blue arrows indicate the sites of covalent attachments to cysteine thiols. The red moieties corresponding to the methyl (MD) and ethyl (ED) substituents.

DMSO, MD, ED, and EM treatments and the FP measurements of CALP and CALP^C319A^ were performed in parallel. Statistics were calculated from three independent experiments using linear mixed effect model in R (package nlme).

To test the hypothesis that the observed loss of affinity is really due to modification at Cys^319^ as opposed to some other chemical effect, we performed equivalent measurements following the treatment of the CALP^C319A^ mutant. Using the same labeling conditions, we observed neither modification (Supplementary Figure S9G–I) nor inhibition of the mutant protein (Supplementary Figure S9C and Table 2). Taken together, our data are consistent with a model in which covalent modification of Cys^319^ is primarily responsible for CALP inhibition mediated by dephostatin compounds.

## Concluding remarks

Although of limited utility as therapeutic scaffolds, MD and ED appear to represent the first examples of small-molecule allosteric regulators of PDZ–peptide interactions. Leu^291^ is one of the residues exhibiting stronger chemical-shift perturbations following covalent attachment of MD than during non-covalent interaction. Leu^291^ is the ‘L’ residue in the GLGI motif, the common PDZ-domain loop that forms critical hydrogen bonds with the peptide main-chain carboxylate. It is also the subject of strong chemical-shift perturbation at MD concentrations that are associated with covalent modification (Figure 5D, red box), and is thus a perfect candidate to mediate conformational cross-talk between the dual ligands. G^290^, G^292^, and I^293^ from the GLGI motif also showed chemical-shift perturbations in both states (Figure 5D, black ovals). Our crystal structure does not show gross displacement of the carboxylate-binding loop, but we have captured a ternary complex that includes the peptide ligand. As a result, mass action may have obscured an alternative conformation of the loop. Taken together, our data support the hypothesis that MD reduces the free energy of an unbound conformation of the loop, rendering the process of peptide binding less favorable thermodynamically.

Intriguingly, MD appears to act not as a reversible allosteric inhibitor, but rather as a covalent allosteric inhibitor. There is a small but growing literature on the development of covalent allosteric enzyme inhibitors. For example, covalent modification on allosteric residue Cys^121^ can inactivate protein tyrosine phosphatase 1B [48]. Non-catalytic residues Cys^296^ and Cys^310^ of AKT were targeted by effective inhibitors that stabilize the inactive conformation of the kinase [49]. Covalent modification of the cysteine residue in oncogenic mutant K-Ras^G12C^ was found to favor GDP over GTP binding in the active site and to specifically prevent binding to the downstream effector protein Raf [50]. Moreover, chemical approaches have been taken to explore the vulnerability of accessible cysteines in cancer therapy [51].

From the perspective of inhibitor development, lessons can be drawn from the kinase inhibitors: covalent allosteric inhibitors have shown increased selectivity and potency [49]. Despite the potential concerns of covalent interactions, there are also advantages: a preference for shallow binding pockets; increased biochemical efficacy; longer duration and lower effective dose due to a negligible off-rate; and increased specificity by avoiding off-target interactions with related peptide-binding sites [52,53].

In any case, by acting allosterically, MD appears to exploit a conformational flexibility that is a common feature of this class of protein–protein interaction modules. Previous studies have shown that PDZ domains can be allosterically structured by their neighbors. For example, the PDZ2 domain of X11/MINT exhibits WT binding specificity only when covalently attached to the PDZ1 domain and to the C-terminal tail of the protein [54]. Similarly, in PSD95 PDZ3, an α-helix appended to the C-terminus does not directly contact the peptide but increases the affinity of peptide binding 21-fold [55]. Spectroscopic studies have identified intramolecular couplings that can provide the basis of such effects [55,56]. Similarly, statistical interactions based on sequence comparisons of 274 eukaryotic PDZ domains suggest a free-energy propagation pathway connecting the peptide-binding cleft to the α1 helix and the other side of the PDZ domain through its core [57]. A similar effect might explain the recent observation that tyrosine phosphorylation on the back side of the PDZ2 and PDZ3 domains of PSD-95 decreases their peptide-binding affinity [58]. The prospect of allosteric inhibition expands the potential target surfaces on such protein–protein interaction domains. In circumventing the relatively flat primary interaction site, this strategy may thus offer new approaches to address a historically elusive class of targets.

## Supporting information

**Figure S1:** CAL-specific RNA interference increases cell-surface and WCL abundance of WT-CFTR

**Figure S2:** The overall screening strategy

**Figure S3:** Ussing-chamber measurements show that MD does not stimulate CFTR chloride currents in CFBE-ΔF cells

**Figure S4:** Hetero-trimer formation

**Figure S5:** Alignments of the models of CALP complexes

**Figure S6:** Mass spectrometry confirms the covalent attachment of MD

**Figure S7:** CALP-MD adduct formation with different MD concentrations under NMR conditions

**Figure S8:** NMR HSQC titration of MD with CALP^C319A^

**Figure S9:** FP measurements of the inhibition effects of MD, ED and EM

## Abbreviations

AS: AlphaScreen
BT: biotin
CAL: CFTR-associated ligand
CALP: PDZ domain of CAL
Cer-CALP: CAL PDZ domain with a Cerulean tag
CF: cystic fibrosis
CFTR: CF transmembrane conductance regulator
Dlg1: discs large 1
ED: ethyl-3,4-dephostatin
EM: N-ethyl-maleimide
FP: fluorescence polarization
F508del: p.Phe508del
HSQC: heteronuclear single quantum coherence
HTS: high-throughput screen
LDH: lactate dehydrogenase
MD: methyl-3,4-dephostatin
MTS: 3-(4,5-dimethylthiazol-2-yl)-5-(3-carboxymethoxyphenyl)-2-(4-sulfophenyl)-2H-tetrazolium
NHERF: Na^+^/H^+^ Exchanger Regulatory Factor
PAINS: pan–assay interference compound
PDZ: PSD-95/Dlg1/ZO-1
PSD-95: post-synaptic density 95
TCEP: tris(2-carboxyethyl)phosphine
