# Correction: Cysteine modification reveals an allosteric inhibitory site on the CAL PDZ domain

**DOI:** 10.1042/BSR20180231_COR

**Published:** 2018-07-06

**Authors:** Yu Zhao, Patrick R. Cushing, David C. Smithson, Maria Pellegrini, Alexandre A. Pletnev, Sahar Al-Ayyoubi, Andrew V. Grassetti, Scott A. Gerber, R. Kiplin Guy, Dean R. Madden

***Bioscience Reports* (2018)** https://doi.org/10.1042/BSR20180231

After acceptance and the publication of the Accepted 3, follow-up experiments revealed unexpected variability in the binding constants obtained by the mixed-*K*_D_ analysis of FP binding data as shown in Table 2 in the Accepted Manuscript. Further investigation using non-reducing SDS–PAGE found previously undetected PDZ–PDZ dimerization triggered by extended incubation with the small-molecule compounds under stringent labeling conditions. This dimerization is not accounted for by our mixed-*K*_D_ model. The final Version of Record instead reports apparent *K*_D_ values determined from FP titrations with PDZ domains treated to minimize PDZ–PDZ dimerization while preserving high levels of small-molecule adduct formation, together with associated changes in text.

This has been approved by the Editorial Board of *Bioscience Reports.*

