# Supplementary material for: Cysteine modifiers suggest an allosteric inhibitory site on the CAL PDZ domain
Source: Biosci Rep. 2018 Jul 6;38(4):BSR20180231. doi: 10.1042/BSR20180231 (PMC6435542; doi:10.1042/BSR20180231)
Supplement: Supplementary file 1 [file bsr20180231_Supp1.pdf]

Supplementary Material for:

Cysteine modifiers suggest an allosteric inhibitory site on the CAL PDZ domain

Yu Zhao[1,*], Patrick R. Cushing[1,*], David C. Smithson[2], Maria Pellegrini[3], Sahar Al-Ayyoubi[1], Andrew V. Grassetti[1], Scott A. Gerber[1,4], R. Kiplin Guy[2] and Dean R. Madden[1, **]

[1]Departments of Biochemistry & Cell Biology and [4]Department of Molecular & Systems Biology, Geisel School of Medicine at Dartmouth, Hanover, NH 03755, USA

[2]Department of Chemical Biology and Therapeutics, St. Jude Children's Research Hospital, Memphis, TN 38105, USA

[3]Department of Chemistry, Dartmouth College, Hanover, NH03755, USA

[*] Equal contribution

[**] Corresponding author: Dean R. Madden, Dept. of Biochemistry & Cell Biology, Geisel School of Medicine, 7200 Vail Building, Hanover, NH 03755 USA. Tel: +1-603-650-1164. Email: drm0001@dartmouth.edu

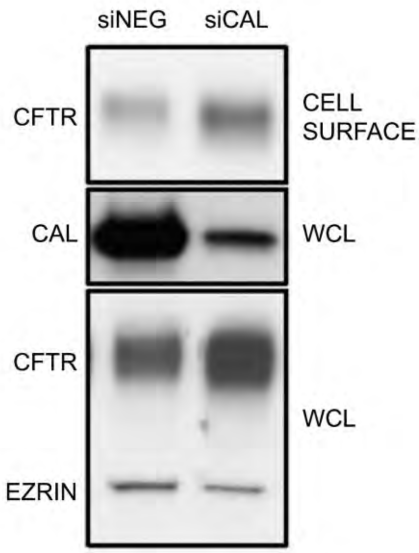

Figure S1: CAL-specific RNA interference increases cell-surface and WCL abundance of WT-CFTR

Polarized CFBE cells expressing WT-CFTR (WT-CFBE cells) were treated with CAL-specific (siCAL) or negative control (siNEG) siRNA. Cells were grown on filters at an air-liquid interface for three days, and then surface proteins were biotinylated. Cells were lysed and biotinylated protein were captured by streptavidin beads and probed to determine CFTR levels. Ezrin: loading control marker. The knockdown efficiency of siCAL was 86 ± 11% (n=3).

Following normalization to an ezrin loading control, surface biotinylated and whole-cell lysate (WCL) CFTR siCAL-treated cells showed statistically significant average increases of 2.33-fold (p = 0.00007) and 1.65-fold (p = 0.00021), respectively, compared to siNEG-treated controls. Statistics were calculated from three independent experiments each with two or four replicates, using a linear mixed-effects model in R (package nlme).

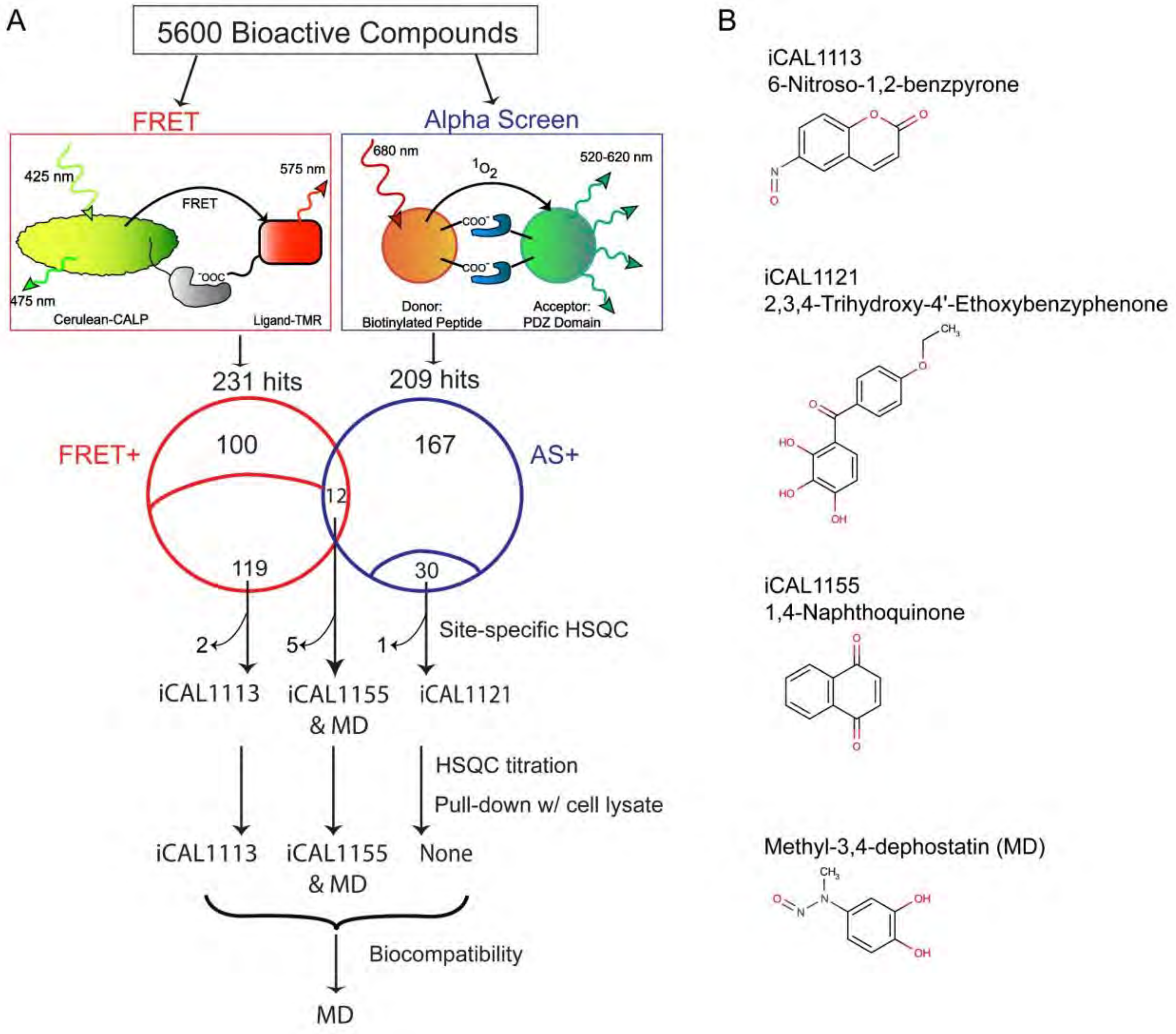
A
5600 Bioactive Compounds
FRET
Alpha Screen
425 nm
575 nm
FRET
475 nm
Cerulean-CALP
Ligand-TMR
680 nm
$^1O_2$
520-620 nm
Donor: Biotinylated Peptide
Acceptor: PDZ Domain
231 hits
209 hits
FRET+
AS+
100
167
12
119
30
Site-specific HSQC
2
5
1
iCAL1113
iCAL1155 & MD
iCAL1121
HSQC titration
Pull-down w/ cell lysate
iCAL1113
iCAL1155 & MD
None
Biocompatibility
MD
B
iCAL1113
6-Nitroso-1,2-benzpyrone
iCAL1121
2,3,4-Trihydroxy-4'-Ethoxybenzyphenone
iCAL1155
1,4-Naphthoquinone
Methyl-3,4-dephostatin (MD)

Figure S2: The overall screening strategy

(A)The flow chart of the screening strategy. In a FRET screen (red box and circle) of the St. Jude bioactive collection (5600 wells), a Cerulean domain fused to CALP acts as a donor to the TMR label attached to reporter peptide iCAL36. Inhibitors reduce the FRET signal measured as the ratio of fluorescence intensities $F_{575\ nm}/F_{475\ nm}$). In the Alpha-Screen (blue box and circle), a biotinylated peptide attached to streptavidin donor beads interacts with polyhistidine-tagged CerCALP attached to NiNTA acceptor beads, permitting proximity-based exchange of singlet oxygen. Inhibitors disrupt the coupling, decrease acceptor-bead emission. Only 12 compounds were identified by both screens, one of which was eliminated as a likely fluorescence artifact. In the NMR secondary screen, 12 $FRET^+/AS^+$ compounds, 119 $FRET^+/AS^-$ and 30 $AS^+/FRET^-$ compounds were tested using single-point NMR ^1H-^{15}N HSQC spectra. Among the compounds that disrupted binding, five from the $FRET^+/AS^+$ set, two from the $FRET^+/AS^-$ set, and one from the $AS^+/FRET^-$ set acted as protein aggregators. Tests for saturable binding and ability to inhibit endogenous CAL in a pull-down assay eliminated an additional compound. Three candidates demonstrated saturable binding and site-specific footprints. Biocompatibility assays validated MD as the primary lead. (B) The chemical identities and schematics of the four HSQC validated site-specific interactors.

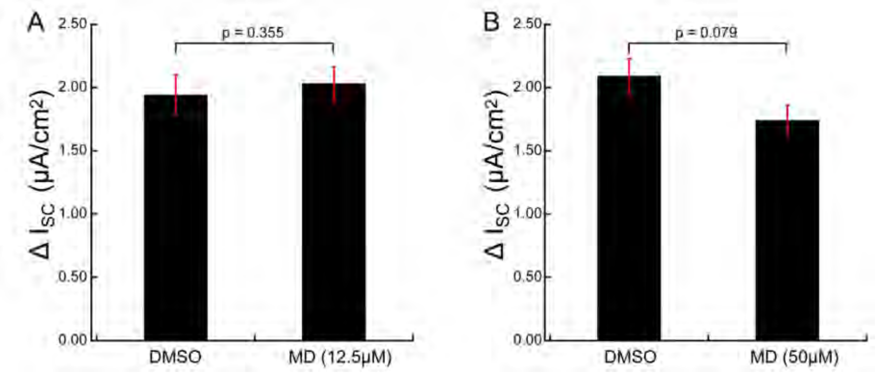

Figure S3. Ussing-chamber measurements show that MD does not stimulate CFTR chloride currents in CFBE-ΔF cells

MD was applied to polarized CFBE-ΔF cells at a final concentration of 12.5 µM (A; n=6) or 50 µM (B, n=8). The change in short-circuit current (I_{SC}) was measured in response to $CFTR_{inh}$172 (ΔI_{SC}). No significant changes were seen in either case, although a trend to lower current was observed at the higher dose.

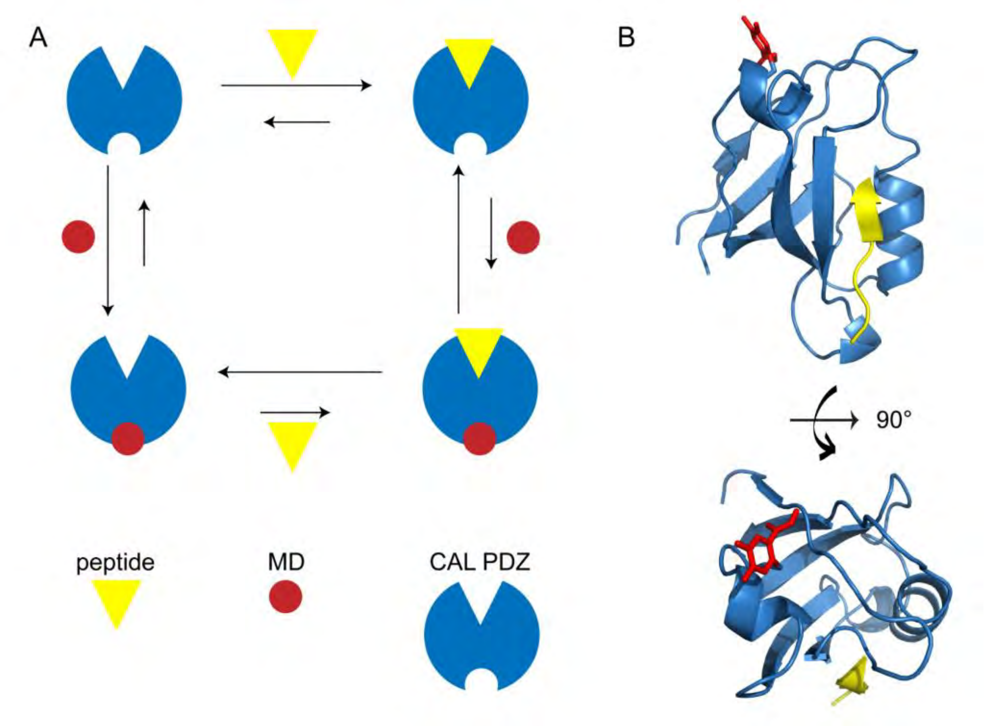

Figure S4. Hetero-trimer formation

(A) Due to the non-competitive mode of inhibition, high concentrations of peptide ligand (yellow triangles) and MD inhibitor (red dots) can drive formation of a hetero-trimer with the CAL PDZ domain (blue notched circle). The hetero-trimer corresponds to the structure determined by co-crystallization in (B). (B) PyMOL views of the hetero-trimer CALP:HPV18E6:MD (PDB ID **5IC3**). CALP (blue); peptide HPV18E6 (yellow); MD (red).

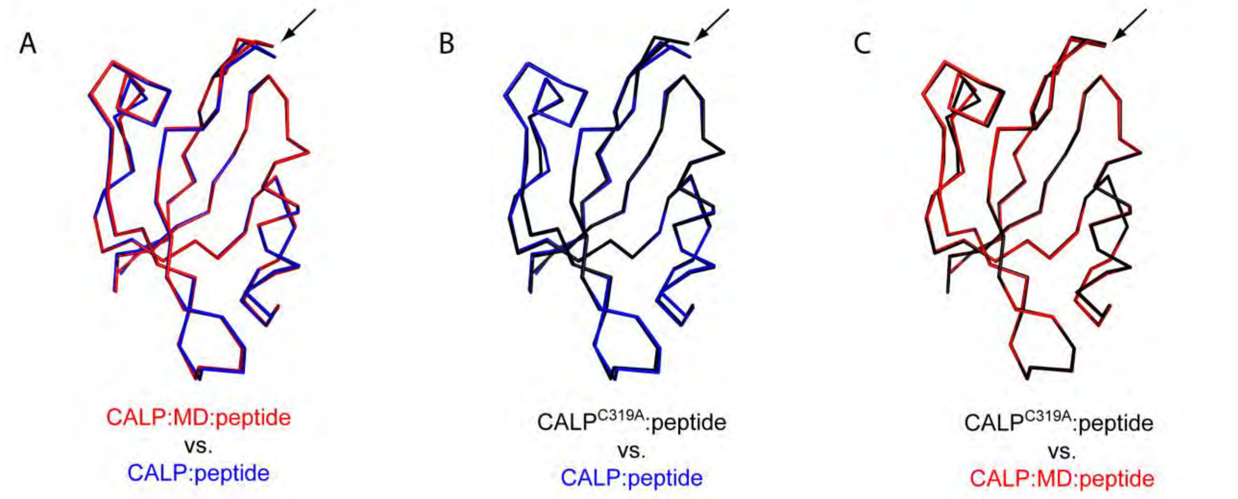

Figure S5. Alignments of the models of CALP complexes

(A) CALP:MD:peptide model **5IC3** (red, chain A) aligned with CALP:peptide model **4JOR** (blue, chain A) (RMSD = 0.213 Å; 504 atoms). Black arrow points to the loop movement seen between the two models.

(B) CALP^{C319A}:peptide model **5K4F** (black, chain A) aligned with CALP:peptide model **4JOR** (blue, chain A) (RMSD = 0.213 Å; 472 atoms). Black arrow points to the loop movement seen between the two models.

(C) CALP^{C319A}:peptide model **5K4F** (black, chain A) aligned with the CALP:MD:peptide model **5IC3** (red, chain A) (RMSD = 0.119 Å; 445 atoms). Black arrow points to the very close overlay of the loops in the two models.

PyMOL was used to perform all the alignments.

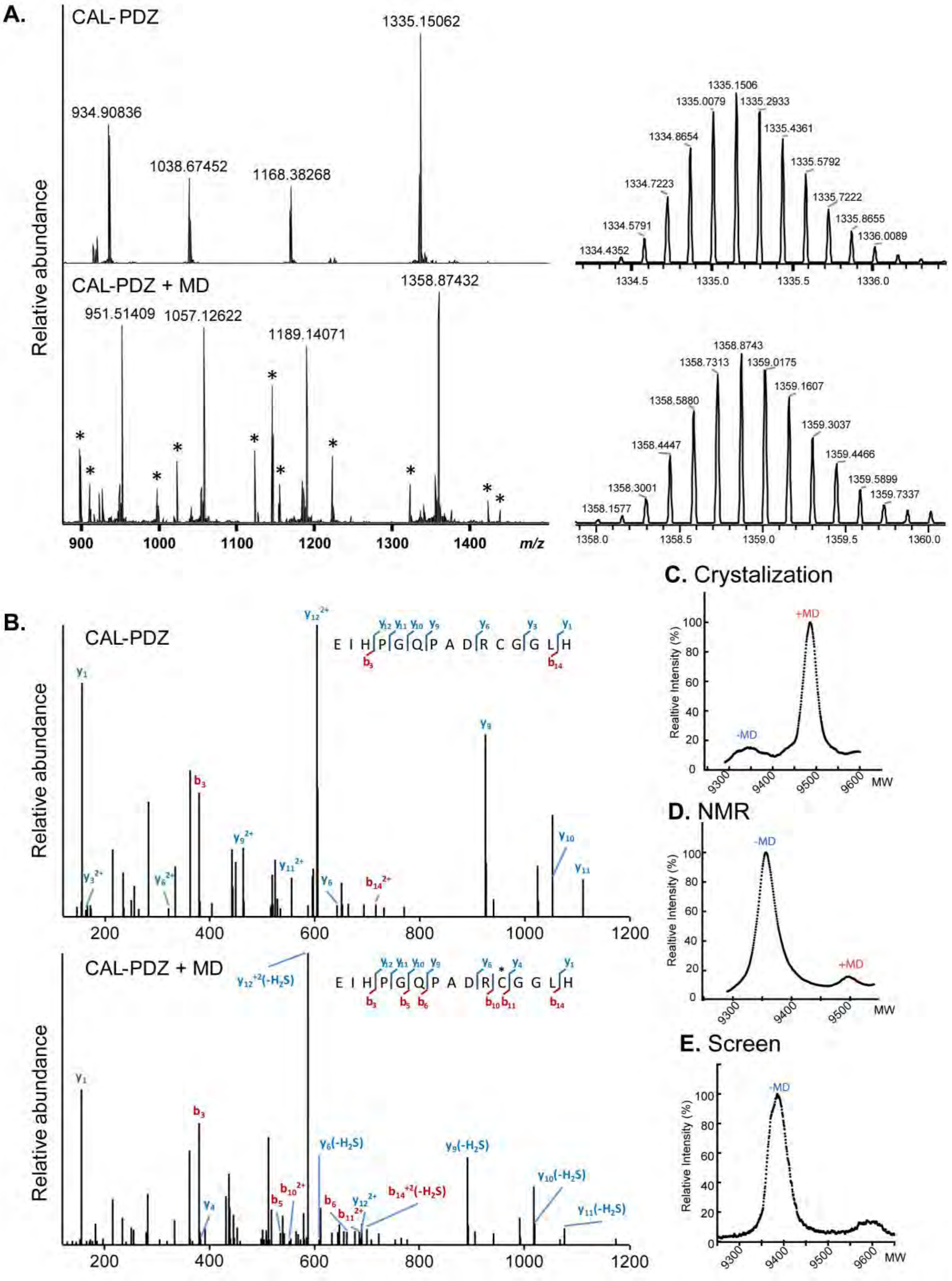
A.
CAL- PDZ
1335.15062
934.90836
1038.67452
1168.38268
CAL-PDZ + MD
1358.87432
951.51409
1057.12622
1189.14071
Relative abundance
900 1000 1100 1200 1300 1400 m/z
1335.1506
1335.0079 1335.2933
1334.8654 1335.4361
1334.7223 1335.5792
1335.7222
1334.5791 1335.8655
1336.0089
1334.4352
1334.5 1335.0 1335.5 1336.0
1358.8743
1358.7313 1359.0175
1358.5880 1359.1607
1358.4447 1359.3037
1359.4466
1358.3001 1359.5899
1359.7337
1358.1577
1358.0 1358.5 1359.0 1359.5 1360.0
B.
CAL-PDZ
E I H P G Q P A D R C G G L H
Relative abundance
200 400 600 800 1000 1200
CAL-PDZ + MD
E I H P G Q P A D R C G G L H
200 400 600 800 1000 1200
C. Crystalization
+MD
-MD
Realtive Intensity (%)
9300 9400 9500 9600 MW
D. NMR
-MD
+MD
Realtive Intensity (%)
9300 9400 9500 MW
E. Screen
-MD
Relative Intensity (%)
9300 9400 9500 9600 MW

Figure S6. Mass spectrometry confirms the covalent attachment of MD

(A) Intact electrospray ionization Orbitrap analysis at 240,000 resolution (FWHM @ 200 *m/z*) shows the intact CAL-PDZ domain by itself (upper) and modified by MD (lower). The left panels show charge states from $(M+H^+)^{7+}$ to $(M+H^+)^{10+}$, ranging from 875 to 1,500 Thomson. The right panels show zoomed views of the $(M+H^+)^{7+}$ isotopic envelopes that illustrate the shift in mass of CAL-PDZ by covalent addition of MD. Asterisks indicate non-specific MD background ions not related to CAL-PDZ ($z < 4$).

(B) Annotated MS^2 spectra are shown for native (upper) or adducted (lower) CAL-PDZ peptides derived from proteinase-K digests and containing Cys^{319} with or without the MD covalent modification. In addition to differences in parent ion mass < 2.5 parts-per-million (ppm) from theoretical, the mass of the modification is identified in the b_{11}^{2+} and y_{12}^{2+} fragment ions from CAL-PDZ + MD. Additionally, the presence of the modification was inferred in fragment ions b_{14}^{2+}, y_6^{2+}, y_9^{2+}, y_{10}^{2+}, y_{11}^{2+}, and y_{12}^{2+} wherein ions consistent with the additional loss of H_2S are observed.

MALDI-TOF confirmed that (C) under crystallization conditions, over 85% of CALP was modified by MD (n=3); (D) under the NMR condition (with 125 µM MD), about 13% of CALP was modified by MD; (E) under FRET screening conditions, CALP was not modified by MD (n=3).

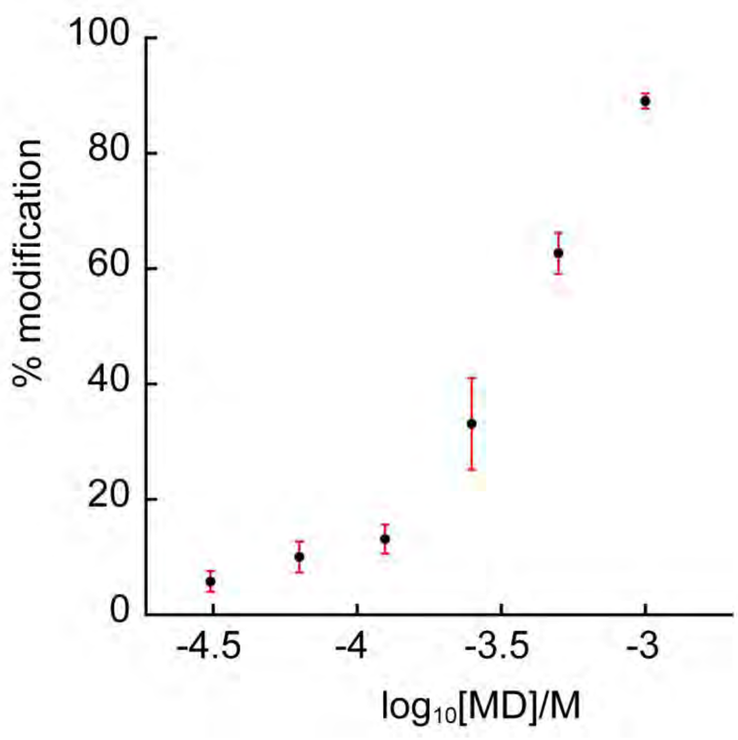

Figure S7. CALP-MD adduct formation with different MD concentrations under NMR conditions

Percentage of CALP-MD adduct formation after co-incubation was measured by MALDI-TOF. Peak mass was determined for signals corresponding to either CALP or CALP-MD (n=3). 50 µM CALP was incubated with different concentrations of MD. Significant CALP-MD adduct formation was observed for MD concentrations at or above 250 µM.

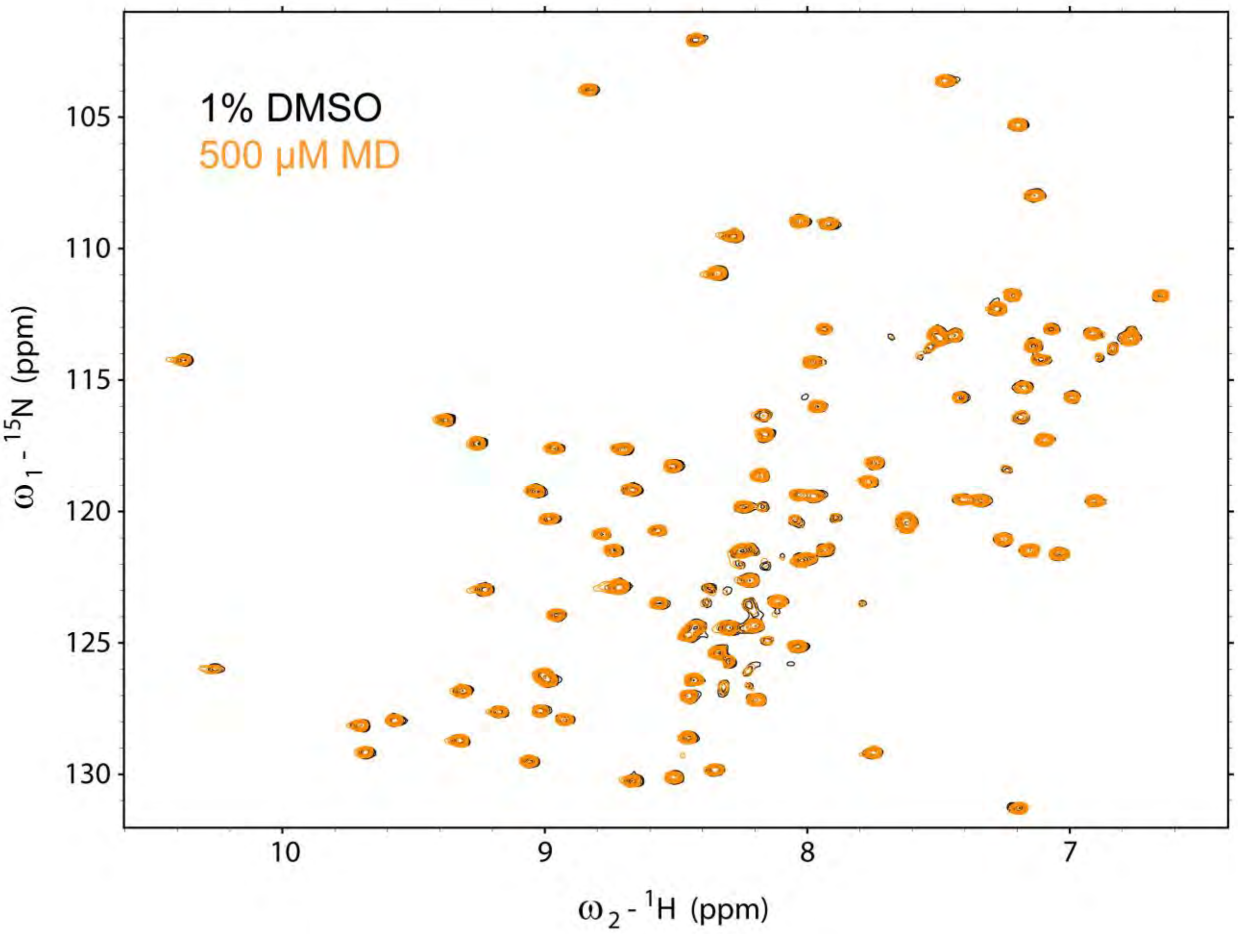

Figure S8. NMR HSQC titration of MD with CALP^{C319A}

HSQC spectra are shown for 50 µM ^{15}N- CALP^{C319A} with 500 µM MD (orange) overlaid with vehicle control (1% DMSO, black). No significant chemical shift perturbations were observed upon addition of 500 µM MD, suggesting that no observable binding occurred between MD and CALP^{C319A} under the condition tested. A two-fold HSQC titration was performed using concentrations of MD between 15 µM and 1 mM, and similar results were observed (data not shown).

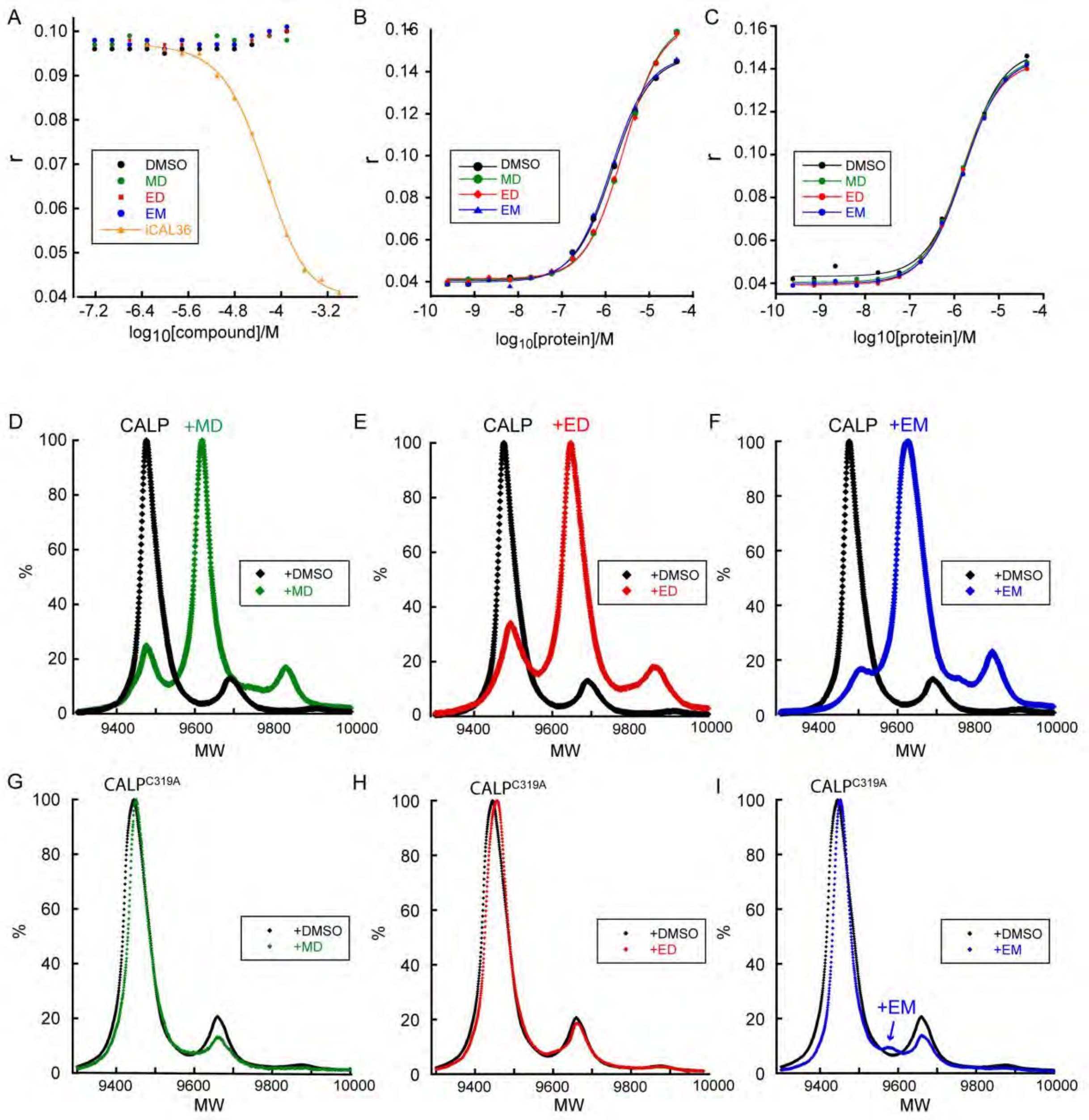

Figure S9. FP measurements of the inhibition effects of MD, ED and EM

(A) FP competition assay to measure the K_I of MD, ED and EM at pH6.8 (25 mM sodium phosphate [pH 6.8], 150 mM sodium chloride, 0.1 mM TCEP, 0.02% sodium azide) that disfavor covalent modification of CAL PDZ (see Figure S6D). DMSO was the negative control; peptide inhibitor iCAL36 was the positive control. The figure represents one of three independent

measurements. (B) FP binding assay of DMSO-, MD-, ED- or EM- treated CALP. The figure represents three independent measurements. (C) FP binding assay DMSO-, MD-, ED- or EM-treated CALP^{C319A}. The figure represents three independent measurements. (D-F) MALDI-TOF curves show the modification status of CALP following incubation with MD (D), ED (E), or EM (F) used in the FP binding assays shown in (B). (G-I) MALDI-TOF curves show the modification status of CALP^{C319A} following incubation with MD (G), ED (H), or EM (I) used in the FP binding assays shown in (C).
